# Development of a quality indicator set for the responsibility-based holistic nursing model: a Delphi study based on the donabedian framework

**DOI:** 10.3389/fmed.2026.1834033

**Published:** 2026-05-29

**Authors:** Xiaoyan Wang, Zheng Chen, Ruonan Chuai, Yue Shen, Cuizhe Yang, Wei Li, Luqin Di

**Affiliations:** Department of Orthopaedic Surgery, The Third Hospital of Hebei Medical University, Shijiazhuang, Hebei, China

**Keywords:** Delphi, indicators, nursing, quality, responsibility-based holistic nursing model

## Abstract

**Background:**

The responsibility-based holistic nursing model is widely implemented in Chinese hospitals. However, a structured quality indicator set for assessing this model is still absent. We aim to develop a standardized quality indicator set, thereby providing a reference for the further implementation of this methodology.

**Methods:**

Using the “structure-process-outcome” quality framework as a theoretical foundation, evaluation indicators for the responsibility-based holistic nursing model were constructed through a literature review and semi-structured interviews with nursing staff. A two-round Delphi survey was performed with 18 nursing experts to determine content validity and achieve consensus. Indicator weights were calculated using the normalized mean importance score method.

**Results:**

The response rates for both sets of correspondence questionnaires were 100%. The expert authority levels were 0.904 and 0.895, and the Kendall’s coefficients of concordance were 0.153 and 0.182, both with *P* < 0.01. The final indicator set comprised 3 primary indicators, 12 secondary indicators, and 50 tertiary indicators.

**Conclusion:**

This study established a preliminary quality indicator set for the responsibility-based holistic nursing model based on the Donabedian framework. Developed with moderate expert consensus, the indicator set offers a reference for evaluating nursing quality in hospitals. However, its practical applicability remains to be validated through clinical practice.

## 1 Introduction

The responsibility-based holistic nursing model is a care-delivery system widely implemented in Chinese hospitals. In this model, a fixed nurse is responsible for a defined group of patients throughout their hospital stay, providing comprehensive, continuous nursing services that include medical care, condition monitoring, treatment assistance, and health guidance. Continuity of care is maintained through organized bedside handovers and collaborative communication among teams.

The effective implementation of the responsibility-based holistic nursing model directly impacts the quality of clinical nursing in hospitals (1, 2). However, a scientific, systematic, and comprehensive set of quality indicators for assessing the implementation of this model remains absent. Developing such an indicator set is essential for the effective implementation of the responsibility-based holistic nursing model and for attaining ongoing quality improvement.

Current research offers a rather constrained examination of evaluation indicators for responsibility-based holistic nursing. The majority of assessment indicators for responsibility-based holistic nursing are generic nursing quality indicators that fail to appropriately reflect the fundamental components of the responsibility-based holistic nursing model, and evaluation dimensions are generally limited (3).

Therefore, this study aims to develop a scientific and practical system of quality indicators for responsibility-based holistic nursing based on Donabedian’s structure-process-outcome framework. Our objective is to offer a reference for objectively evaluating the implementation of the responsibility-based holistic nursing model in hospital inpatient wards.

## 2 Materials and methods

### 2.1 Establish a research team

The team consists of six members: one nursing department director responsible for nursing quality, two head nurses from inpatient areas, two clinical nurses, and one postgraduate nursing student. The study team’s responsibilities include conducting a literature review, developing initial evaluation indicators, selecting consulting experts, designing and distributing expert consultation questionnaires, as well as organizing, summarizing replies, and conducting statistical analysis.

This study was approved by the Ethics Committee of the Third Hospital of Hebei Medical University (No. 2023-065-1). The purpose and methods of the study were explained to all participants, and only those who voluntarily consented to participate were included after signing an informed consent form. Participants could withdraw from the study at any time without any consequences, and all collected data would be used solely for research purposes.

### 2.2 Formulate a preliminary evaluation checklist

#### 2.2.1 Comprehensive literature and policy review

An integrative review of the literature and relevant policy documents was conducted to generate a preliminary pool of candidate quality indicators. The team members searched the following databases: China National Knowledge Infrastructure (CNKI), Wanfang database, VIP database, and PubMed. We used “responsibility-based holistic nursing,” “nursing quality indicator,” and “quality evaluation” as keywords. The search covers the period from the establishment of the databases to June 30, 2025. Inclusion criteria: ① The research topic is related to the overall quality evaluation of the responsibility-based holistic nursing model; ② The language is either Chinese or English. Exclusion criteria: duplicate inclusion, inability to obtain full text. In addition to academic databases, key national policy documents and clinical practice guidelines were reviewed to identify quality standards and indicators specific to the responsibility-based holistic nursing model. All retrieved items were screened for relevance, and candidate indicators were extracted to form the initial indicator pool.

#### 2.2.2 Semi-structured interviews

Based on the integrative literature and policy review, 9 nurse managers were selected for semi-structured interviews. The interview framework included the following questions: ① What do you consider the principal aspects and challenges in the execution of the responsibility-based holistic nursing model? ② What suggestions do you have for the implementation of the responsibility-based holistic nursing model? ③ What criteria do you think can be used to evaluate the efficacy of responsibility-based holistic nursing? ④ If you are assigned to develop evaluation indicators for the quality of responsibility-based holistic nursing, which specific indicators would you prioritize? Which quality evaluation indicators would you prioritize for responsibility-based holistic nursing? ⑤ How would you obtain data on these indicators?

All interviews were conducted by a fixed team member, audio-recorded with consent, and transcribed verbatim within 24 h. Transcripts were analyzed using Colaizzi’s method by two independent researchers. Data saturation was reached when three consecutive interviews yielded no additional codes.

Based on a review of the literature, interview data, and national policy considerations, preliminary quality assessment indicators for responsibility-based holistic nursing have been formulated. These include 3 primary, 12 secondary, and 55 tertiary indicators.

### 2.3 Expert consultation

#### 2.3.1 Questionnaire development

The questionnaire included four sections: ① Instructions for the questionnaire, detailing the study context, objectives, completion guidelines, submission deadline, and contact details. ② Personal information of experts, such as age, educational background, and professional title. ③ Evaluation indicators: A 5-point Likert scale evaluated the importance of each item, with 1 indicating “very unimportant” and 5 meaning “very important.” Each item has a notes section for experts to provide critiques, recommend improvements, or propose additions or deletions. ④ Expert self-assessment form, detailing the basis for expert evaluation and the level of familiarity with each subject.

#### 2.3.2 Expert selection

Experts in nursing management and clinical nursing at Grade A tertiary hospitals were invited. Inclusion criteria for experts: ① A bachelor’s degree or above; ② At least 10 years of nursing management experience; ③ An associate senior professional title or higher; ④ Voluntary participation in this study with a commitment to its completion.

#### 2.3.3 Implementation of consultation

The research team sent questionnaires to experts via email from February to April 2025, requesting their return within 2 weeks. Indicators were kept if the average importance score was greater than 3.5, the coefficient of variation was less than 25%, and the full-score rate was greater than 20% (4). Based on expert opinions, indicators were modified, added, or deleted to develop the next round of questionnaires.

The consultation ended when expert opinions became consistent. This study performed two rounds of expert consultation.

### 2.4 Statistical analysis

Data analysis was conducted using Excel 2016 and SPSS 25.0. Measurement data, including expert information and indicator scores, were presented as means, standard deviations, and percentages. The degree of expert engagement was measured by questionnaire response rate, with higher rates indicating greater engagement (5). The degree of authority was assessed using the expert authority coefficient, where a coefficient above 0.7 signifies dependable consultation results (6). The degree of agreement among expert opinions was expressed using Kendall’s coefficient of concordance and the coefficient of variance, reflecting the consistency of experts’ views on each indicator. Indicator weights were calculated using the normalized mean importance score method. The weight of each indicator was derived as the proportion of its mean expert rating relative to the sum of mean ratings within its respective parent category. A *P*-value < 0.05 was considered statistically significant.

## 3 Results

### 3.1 Basic information of experts

This study finally encompassed 18 experts from three provinces in China. Their ages ranged from 38 to 56, with an average age of (48.67 ± 5.49) years; their years of employment ranged from 14 to 37, with an average work experience of (27.50 ± 6.78) years. Four experts (22.22%) possessed a master’s degree, while 14 (77.78%) had a bachelor’s degree; ten experts (55.56%) attained an associate senior title, and 8 (44.44%) had a senior title.

### 3.2 Experts’ positivity and authority

In both the first and second rounds, 18 questionnaires were distributed, all of which were returned as valid, resulting in a 100% valid response rate. During the first round, 10 experts (55.6%) provided revision suggestions. The familiarity coefficient (Cs) was 0.847, the judgment basis coefficient (Ca) was 0.961, and the authority coefficient (Cr) was 0.904. In the second round, 5 experts (27.8%) provided revision suggestions, with Cs at 0.833, Ca at 0.956, and Cr at 0.895.

### 3.3 Degree of coordination among expert opinions

During the first round, the coefficient of variation ranged from 0 to 0.324, while Kendall’s coefficient of concordance was 0.153 (χ^2^ = 190.407, *P* < 0.01). During the second round, the coefficient of variation ranged from 0 to 0.227, and Kendall’s coefficient of concordance was 0.182 (χ^2^ = 219.608, *P* < 0.01).

Kendall’s coefficient of concordance was also calculated using only the indicators retained after each round of screening to better reflect consensus on the core items. For these retained indicators, Kendall’s coefficient of concordance was 0.182 in the first round (χ^2^ = 219.608, *P* < 0.01) and 0.181 in the second round (χ^2^ = 208.032, *P* < 0.01). Although Kendall’s W values were low, they showed minimal change between the two rounds. And the coefficient of variation for most retained indicators had stabilized or decreased between the two rounds, suggesting that further rounds would not change the expert panel’s collective opinion. The Delphi process was therefore ended after two rounds. Expert ratings from the second round were used to evaluate content validity. The 50 tertiary indicators have a Scale-level Content Validity Index of 0.98.

### 3.4 Results of expert consultation

After survey collection, the research team amended the indicators in accordance with expert opinions and the established screening criteria.

First round of expert consultation: one secondary indicator, “management regulations,” was deleted, and the three tertiary indicators under it were moved to the secondary indicator “position management.” Five tertiary indicators were deleted, including “Appropriate bed-to-nurse ratio,” “Appropriate nurse tier allocation,” “Correct usage rate of patient-controlled analgesia pumps,” and “implementation rate of Internet Plus Healthcare.” One secondary indicator and four tertiary indicators were modified, such as changing “material allocation” to “support system” and “sufficient number of PDA and mobile nursing carts” to “reasonable configuration of work hardware.” Meanwhile, one secondary indicator and three tertiary indicators were added, such as “nursing management” and “standardization rate of basic technical operations for nurses.” Ultimately, 3 primary indicators, 12 secondary indicators, and 53 tertiary indicators entered the second round of expert inquiry.

Second round of expert consultation: Three tertiary indicators were deleted, including “patient satisfaction with pain management,” “implementation rate of discharge follow-up,” and “pass rate of special inspection for high-quality nursing services.”

The finalized quality indicator set for the responsibility-based holistic nursing model comprises 3 primary indicators, 12 secondary indicators, and 50 tertiary indicators, as presented in Table 1.

**TABLE 1:** Quality evaluation indicators for the responsibility-based holistic nursing model.

Item	Full-score rate (%)	Importance ($\bar{\text{x}}$ ± s)	CV	Weight	Combined weight	I-CVI
I Structural indicators	100	4.78 ± 0.43	0.089	0.327		
I-1 Human resource allocation	100	4.67 ± 0.49	0.104	0.162		
I-1-1 Nurses’ qualifications meet the access standards	94.4	4.61 ± 0.61	0.132	0.327	0.017	0.944
I-1-2 Reasonable nurse-to-patient ratio	100	4.72 ± 0.46	0.098	0.335	0.018	1
I-1-3 Establish a flexible staff scheduling mechanism	100	4.78 ± 0.43	0.089	0.339	0.018	1
I-2 Physician-nurse collaboration	100	4.89 ± 0.32	0.066	0.170		
I-2-1 Daily rounds conducted by responsible nurses and attending physicians	100	4.83 ± 0.38	0.079	0.334	0.019	1
I-2-2 Collaborative patient management by physicians and nurses	100	4.78 ± 0.43	0.089	0.331	0.018	1
I-2-3 Physicians and nurses have timely and smooth communication channels	100	4.83 ± 0.38	0.079	0.334	0.019	1
I-3 Position management	94.4	4.67 ± 0.59	0.127	0.162		
I-3-1 Clearly defined responsibilities for all positions	100	4.83 ± 0.38	0.079	0.145	0.008	1
I-3-2 Standardized and reasonable work procedures for each position	100	4.83 ± 0.38	0.079	0.145	0.008	1
I-3-3 Clear work standards for each position	100	4.83 ± 0.38	0.079	0.145	0.008	1
I-3-4 Effective implementation of bed assignment management	94.4	4.78 ± 0.55	0.115	0.144	0.008	0.944
I-3-5 Holistic patient management by nurses	100	4.94 ± 0.24	0.048	0.149	0.008	1
I-3-6 Stratified management of nurses	100	4.61 ± 0.50	0.109	0.139	0.007	1
I-3-7 Reasonable allocation of auxiliary shifts	88.9	4.39 ± 0.69	0.159	0.132	0.007	0.889
I-4 Support systems	100	4.72 ± 0.46	0.098	0.164		
I-4-1 Rational hardware provision	100	4.72 ± 0.46	0.098	0.494	0.027	1
I-4-2 Rational software provision	100	4.83 ± 0.38	0.079	0.506	0.027	1
I-5 Quality management	100	4.94 ± 0.24	0.048	0.172		
I-5-1 Clear quality control system with defined rules	100	4.78 ± 0.43	0.089	0.494	0.028	1
I-5-2 Continuous improvement in nursing quality	100	4.89 ± 0.32	0.067	0.506	0.028	1
I-6 Training and assessment	100	4.89 ± 0.32	0.066	0.170		
I-6-1 Layered training	100	4.67 ± 0.49	0.104	0.332	0.018	1
I-6-2 Training content coverage at each level	100	4.67 ± 0.49	0.104	0.332	0.018	1
I-6-3 Pass rate of training assessments at each level	100	4.72 ± 0.46	0.098	0.336	0.019	1
II Process indicators	100	4.94 ± 0.24	0.048	0.338		
II-1 Patient management	100	4.94 ± 0.24	0.048	0.336		
II-1-1 Qualification rate of basic nursing care	100	4.89 ± 0.32	0.066	0.069	0.008	1
II-1-2 Standardized management rate of tubing	100	5.00	0	0.070	0.008	1
II-1-3 Adequacy of complication prevention measures	100	5.00	0	0.070	0.008	1
II-1-4 Intravenous access meets the intravenous therapy standards	100	5.00	0	0.070	0.008	1
II-1-5 Nursing documentation compliance rate	100	4.83 ± 0.38	0.079	0.068	0.008	1
II-1-6 Accurate patient risk assessment rate	100	4.89 ± 0.32	0.066	0.069	0.008	1
II-1-7 Implementation rate of patient risk measures	100	4.94 ± 0.24	0.048	0.069	0.008	1
II-1-8 Nurses are aware of patients’ general information	100	4.72 ± 0.46	0.098	0.066	0.008	1
II-1-9 Nurses are aware of patients’ physical conditions (mental status, vital signs, skin condition, pain, diet, sleep, bowel and bladder function, abnormal lab results)	100	4.94 ± 0.24	0.048	0.069	0.008	1
II-1-10 Nurses are aware of patients’ specialized conditions (peripheral circulation, drainage status, reactions after surgery/tests/medication)	100	4.94 ± 0.24	0.048	0.069	0.008	1
II-1-11 Education rate of disease-related knowledge	100	4.67 ± 0.49	0.104	0.065	0.007	1
II-1-12 Nurses can provide appropriate assistance based on patients’ physiological, psychological, and social needs	100	4.61 ± 0.50	0.109	0.065	0.007	1
II-1-13 Respect patients, protect patient privacy, and demonstrate humanistic care	100	4.67 ± 0.49	0.104	0.065	0.007	1
II-1-14 Patients know their responsible nurse	77.8	4.27 ± 0.83	0.193	0.060	0.007	0.778
II-1-15 *Consultation rate of patients or their families at the nursing station	72.2	4.00 ± 0.91	0.227	0.056	0.006	0.722
II-2 Ward management	100	4.83 ± 0.38	0.079	0.328		
II-2-1 Ward maintains quietness, cleanliness, and fresh air	94.4	4.56 ± 0.62	0.135	0.326	0.036	0.944
II-2-2 Ward facilities are in good and ready condition	100	4.72 ± 0.46	0.098	0.337	0.037	1
II-2-3 Standardized terminal disinfection in the ward	100	4.72 ± 0.46	0.098	0.337	0.037	1
II-3 Nursing management	100	4.94 ± 0.24	0.048	0.336		
II-3-1 Compliance rate of nurses’ basic technical operations	100	5.00	0	0.339	0.038	1
II-3-2 Compliance rate of nurses’ specialty operations	100	4.94 ± 0.24	0.048	0.334	0.038	1
II-3-3 Nursing scheduling reflects holistic nursing care	100	4.83 ± 0.38	0.079	0.327	0.037	1
III Outcome indicators	100	4.89 ± 0.32	0.066	0.335		
III-1 Satisfaction	100	4.67 ± 0.49	0.104	0.330		
III-1-1 Patient satisfaction with nursing care	100	4.83 ± 0.38	0.079	0.257	0.028	1
III-1-2 Patient satisfaction with the ward environment	88.9	4.50 ± 0.71	0.157	0.240	0.026	0.889
III-1-3 Physician satisfaction with nursing staff	100	4.56 ± 0.51	0.112	0.243	0.027	1
III-1-4 Nurses’ satisfaction with their professional environment and career development	100	4.89 ± 0.32	0.066	0.260	0.029	1
III-2 Quality monitoring	100	4.94 ± 0.24	0.048	0.349		
III-2-1 *Incidence of adverse events	100	4.94 ± 0.24	0.048	0.341	0.040	1
III-2-2 Timeliness of detection of clinical changes	94.4	4.83 ± 0.51	0.106	0.333	0.039	0.944
III-2-3 *Incidence of complications in patients	94.4	4.72 ± 0.57	0.122	0.326	0.038	0.944
III-3 Patient self-management	100	4.55 ± 0.51	0.112	0.321		
III-3-1 Patient compliance with functional exercise	100	4.83 ± 0.38	0.079	0.503	0.054	1
III-3-2 Patient compliance rate with health education	100	4.78 ± 0.43	0.089	0.497	0.053	1

CV, coefficient of variation; I-CVI, item-level content validity index, calculated as the proportion of experts rating the item as 4 or 5 on a 5-point Likert scale (7). “Combined Weight” applies exclusively to tertiary indicators and is computed as the product of the corresponding primary, secondary, and tertiary weights. CV, 0 reflects unanimous expert consensus, which is typically observed for items mandated by national nursing policy or considered fundamental to clinical quality. *Indicators marked with an asterisk (*) are those for which lower values represent better quality. For all other indicators, higher values represent better quality.

## 4 Discussion

### 4.1 This study developed a preliminary indicator set for the responsibility-based holistic nursing model

The three-dimensional “structure-process-outcome” framework, proposed by Donabedian in 1966, is now widely used in nursing quality assessment (8, 9). This study uses this framework as its theoretical foundation, conducts a comprehensive evaluation and synthesis of relevant literature from both domestic and international sources, and incorporates insights from clinical experience within our institution. Through semi-structured interviews and group discussions, a preliminary draft has been developed, thereby ensuring the scientific rigor of the indicator system.

The selection of experts is important in Delphi studies, as it directly influences the reliability of consultation outcomes (10). This study selected 18 experts with extensive clinical and managerial experience from 18 Grade A tertiary hospitals across three provinces in China. Among these individuals, 22.22% held master’s degrees or higher, all had at least 14 years of professional experience, and held associate senior or higher professional titles. They had extensive academic knowledge and practical experience in their fields, making their opinions extremely representative. The response rates for both rounds of consultation questionnaires in this study were 100%.

Moreover, 55.6 and 27.8% of experts recommended adjustments in the two rounds, indicating significant engagement with the study. The expert authority levels in the two rounds were 0.904 and 0.895, respectively, reflecting a high level of authority. Kendall’s coefficient of concordance was 0.153 and 0.182 (both *P* < 0.01), with a modest increase in the second round. This indicates that experts reached moderate consensus after two rounds of consultation. Consequently, This study developed a preliminary indicator set for the responsibility-based holistic nursing model. However, further validation is required.

### 4.2 Analysis of the content of quality evaluation indicators for the responsibility-based holistic nursing model

This study was developed based on previous research and the literature review. It is policy-focused and takes into account the specific characteristics of responsibility-based holistic nursing, making it more practical and targeted.

#### 4.2.1 Close collaboration among healthcare professionals and the improvement of the nurse support system are vital for the effective implementation of the responsibility-based holistic nursing model

The structure indicators included organizational variables that enable the advancement of nursing practice and the environment under which care is provided. Structural quality underpins the execution of responsibility-based holistic nursing (11). Previous nursing quality evaluation systems have primarily focused on structural factors related to human resources, systems, environment, and training (12). The structural indicators developed in this study encompass 6 secondary indicators: human resource allocation, physician-nurse collaboration, position management, support systems, quality management, and training and assessment, along with 20 tertiary indicators. Two new variables, “physician-nurse collaboration” and “support system,” have been incorporated in contrast to the previous study. Physician-nurse collaboration extends beyond basic cooperation; it involves various aspects, including coordinated doctors-nurses rounds, collaborative patient management, and effective communication routes. The objective is to break down communication barriers and improve the effectiveness and quality of diagnosis and treatment. Through information exchange, doctors and nurses can improve diagnostic accuracy and treatment effectiveness, thereby converting medical decisions into tailored nursing actions. This can enhance nurses’ critical thinking abilities and guarantee patient safety. Effective communication between physicians and nurses can improve nursing quality and prevent medical errors (13). In this study, the coefficient of variation for the “physician-nurse collaboration” indicator was 0.066, indicating unanimous experts recognition of its importance. Another indicator, “support system,” refers to the software and hardware configurations that enable the functionality of the responsibility-based holistic nursing care model. Software includes optimized workflows and management systems, while hardware contains sufficient mobile nursing carts, PDAs, and other essential equipment. An efficient support system enables the relocation of nursing stations, liberating nurses from indirect care tasks, including frequent supply retrieval and running back and forth for communication. This maximizes the time available for direct patient care, thereby creating favorable conditions for the smooth implementation of the responsibility-based holistic nursing model.

#### 4.2.2 Patient management is an important aspect of process indicators, and it reinforces the quality of responsibility-based holistic nursing

Process quality evaluation is a core element in assessing nursing quality (14). Considering the essential factors and challenges in the execution of the responsibility-based holistic nursing, 21 tertiary indicators were formulated across three domains: patient management, ward management, and nursing management, which can accurately reflect process quality. The coefficients of variation for the patient management and nursing management indicators were the lowest, at 0.048 each.

Effective patient management is essential for improving the quality of responsibility-based holistic nursing. Nurses must not only execute routine tasks but also demonstrate systematic clinical thinking, enabling them to effectively evaluate and address patient treatment needs and potential dangers, while fostering a greater sense of autonomy and control over their work. The current proficiency in clinical thinking abilities among nurses varies (15), posing significant challenges to the systematic execution of patient care in nursing practice. This study refines patient management by translating it into specific, observable behavioral standards to standardize nursing practice. For example, the indicator “nurses’ awareness of abnormal laboratory test results” aims to evaluate nurses’ grasp of patients’ problems. Meanwhile, a decrease in “family members consulting at the nursing station” indirectly reflects nurses’ management capabilities. Through proactive assessments and the “bedside work system,” nurses can address potential patient issues, reduce unnecessary family consultations, and improve their sense of autonomy over their work.

In addition, the level of nursing technical abilities is a vital element in implementing the responsibility-based holistic nursing model. Therefore, this study offers a standardized rate indicator for nurses’ operational skills to control their professional proficiency.

#### 4.2.3 Focus on patient outcomes: evaluating the comprehensive effectiveness and value of the responsibility-based holistic nursing model

The outcome indicators reflect the overall effectiveness of the service, encompassing 3 secondary indicators: satisfaction, quality monitoring, and patient self-management, along with 9 tertiary indicators. Patient satisfaction serves as a direct assessment of nursing quality (16). This study assesses the impact of the responsibility-based holistic nursing model from multiple perspectives, including patients, physicians, and nurses, thereby providing diverse evidence for a comprehensive understanding of the model’s implementation efficacy. Quality monitoring includes nursing-sensitive indicators such as adverse events, which directly reflect nursing quality and indicate the safety and efficacy of clinical care; therefore, this study uses it as an objective basis for evaluating the quality of the responsibility-based holistic nursing model. Additionally, this study includes patients’ self-management capabilities as an outcome measure to improve the effectiveness of nursing interventions on health outcomes and to encourage patients to translate disease knowledge into consistent health behaviors.

## Limitation

This study also has certain limitations. Firstly, the expert included only senior nursing managers from tertiary Grade A hospitals in three provinces This may lessen the representative of our results. Secondly, we used screening thresholds (mean importance >3.5, CV <25%, full-score rate >20%) based on previous Delphi studies. We did not test other cut-off values in a sensitivity analysis. Future research could try different thresholds to see if the indicator set changes. Meanwhile, the weighted indicator set has not yet been tested in real clinical settings. Its feasibility, reliability between raters, and practical value still need to be confirmed through future studies. Besides, content validity was assessed using 5-point importance scale rather than the standard 4-point relevance scale. This tends to inflate the index. Future studies could employ a separate relevance rating scale to enable more rigorous content validity assessment.

## 5 Conclusion

Quality evaluation is an essential method for improving nursing quality. Based on the Delphi method, this study has developed a responsibility-based holistic nursing quality evaluation system comprising 3 primary indicators, 12 secondary indicators, and 50 tertiary indicators. The indicator set provides a structured foundation for quality assessment in this context. In the future, it will be applied in clinical practice to further explore its scalability and practicality.

## Data Availability

The original contributions presented in this study are included in this article/supplementary material, further inquiries can be directed to the corresponding authors.
